# Pediatric airway reconstruction: results after implementation of an airway team in Brazil^☆^

**DOI:** 10.1016/j.bjorl.2018.10.011

**Published:** 2018-12-11

**Authors:** Rebecca Maunsell, Nayara Soares Lacerda, Luciahelena Prata, Marcelo Brandão

**Affiliations:** a Universidade Estadual de Campinas (Unicamp), *Hospital das Clínicas*, Divisão de Otorrinolaringologia, Campinas, SP, Brazil; b Universidade Estadual de Campinas (Unicamp), *Hospital das Clinicas*, Departamento de Pediatria, Unidade de Terapia Intensiva Pediátrica, Campinas, SP, Brazil

**Keywords:** Laryngotracheal reconstruction, Subglottic stenosis, Pediatric airway, Tracheostomy, Reconstrução laringotraqueal, Estenose subglótica, Via Aérea Pediátrica, Traqueostomia

## Abstract

**Introduction:**

Since development of pediatric intensive care units, children have increasingly and appropriately been treated for complex surgical conditions such as laryngotracheal stenosis. Building coordinated airway teams to achieve acceptable results is still a challenge.

**Objective:**

To describe patient demographics and surgical outcomes during the first 8 years of a pediatric airway reconstruction team.

**Methods:**

Retrospective chart review of children submitted to open airway reconstruction in a tertiary university healthcare facility during the first eight years of an airway team formation.

**Results:**

In the past 8 years 43 children underwent 52 open airway reconstructions. The median age at surgery was 4.1 years of age. Over half of the children (55.8%) had at least one comorbidity and over 80% presented Grade III and Grade IV subglottic stenosis. Other airway anomalies occurred in 34.8% of the cases. Surgeries performed were: partial and extended cricotracheal resections in 50% and laryngotracheoplasty with anterior and/or posterior grafts in 50%. Postoperative dilatation was needed in 34.15% of the patients. Total decannulation rate in this population during the 8-year period was 86% with 72% being decannulated after the first procedure. Average follow-up was 13.6 months. Initial grade of stenosis was predictive of success for the first surgery (*p* = 0.0085), 7 children were submitted to salvage surgeries. Children with comorbidities had 2.5 greater odds (95% CI 1.2–4.9, *p* = 0.0067) of unsuccessful surgery. Age at first surgery and presence of other airway anomalies were not significantly associated with success.

**Conclusions:**

The overall success rate was 86%. Failures were associated with higher grades of stenosis and presence of comorbidities, but not with patient age or concomitant airway anomalies.

## Resumo

**Introdução:**

Com o desenvolvimento de unidades de terapia intensiva pediátrica, o tratamento de crianças para situações cirúrgicas complexas, como a estenose laringotraqueal, tem sido cada vez mais adequado. Montar equipes coordenadas de via aérea para alcançar resultados aceitáveis ainda é um desafio.

**Objetivo:**

Descrever os dados demográficos e os resultados cirúrgicos dos pacientes durante os primeiros oito anos de uma equipe de reconstrução de via aérea pediátrica.

**Método:**

Revisão retrospectiva de prontuários de crianças submetidas à reconstrução aberta de via aérea em uma unidade de saúde universitária de nível terciário durante os primeiros oito anos de desenvolvimento de uma equipe de vias aéreas.

**Resultados:**

Nos últimos 8 anos, 43 crianças foram submetidas a 52 reconstruções abertas de vias aéreas. A mediana de idade na cirurgia foi de 4,1 anos. Mais da metade das crianças (55,8%) apresentavam pelo menos uma comorbidade e mais de 80% apresentavam estenose subglótica Grau III e Grau IV. Outras anomalias das vias aéreas ocorreram em 34,8% dos casos. As cirurgias feitas foram: ressecções cricotraqueais parciais e estendidas em 50% e laringotraqueoplastia com enxertos anterior e/ou posterior em 50%. A dilatação pós-operatória foi necessária em 34,15% dos pacientes. A taxa de decanulação total nesta população durante o período de 8 anos foi de 86%, com 72% dos pacientes decanulados após o primeiro procedimento. O seguimento médio foi de 13,6 meses. O grau inicial de estenose foi preditivo de sucesso para a primeira cirurgia (*p* = 0,0085), 7 crianças foram submetidas a cirurgias de resgate. Crianças com comorbidades apresentaram uma probabilidade 2,5 vezes maior (IC95% 1,2-4,9, *p* = 0,0067) de cirurgias sem sucesso. A idade na primeira cirurgia e presença de outras anomalias das vias aéreas não foram significantemente associadas ao sucesso.

**Conclusões:**

A taxa global de sucesso foi de 86%. As falhas foram associadas a graus maiores de estenose e a presença de comorbidades, mas não com a idade do paciente ou anomalias concomitantes das vias aéreas.

## Introduction

Treatment of congenital and acquired airway pathology in children is still a challenge. Multiple specialties can and should be involved to assure that a whole range of treatment options from endoscopic balloon dilatation to open airway surgery through cervical and thoracic approaches can be performed according to each patient's need. In the recent past in this country children with laryngeal stenosis underwent tracheostomy and allowed to grow into adulthood before a surgical treatment was offered. Since development of pediatric intensive care units, children have increasingly and appropriately been treated for complex surgical situations such as laryngotracheal stenosis.

Open airway surgeries have become more successful even in very small children in the past 30 years.^1^, ^2^, ^3^, ^4^, ^5^ Acknowledgment of the particular needs and care in the preoperative and postoperative period as well as better development of technical skills to perform these surgeries in small airways has been paramount to achieve high success rates and low mortality. Nowadays success rates are comparable to reconstructive surgeries performed in adults.^6^ Reconstructive laryngotracheal surgeries in small airways are technically more demanding since edema and inflammation of small airways may delay extubation and demand coordinated postoperative care with close interaction between surgeons and pediatric intensive care teams to avoid complications. Difficulties in establishing a functional team where surgeons, anesthesiologists, pediatricians and nurses comprehend the importance of all the steps involved in reconstructive airway surgery from surgical instrumentation to non-invasive ventilation techniques are the main reasons why this is usually accomplished in centers performing a significant number of surgeries per year.^2^, ^3^

The use of inappropriate stents, double-stage surgery, age, comorbidities, presence of multiple airway anomalies and complex stenosis are usually listed as causes for poor results.^7^ Laryngotracheoplasty (LTP) consists of the expansion of the airway lumen with cartilage grafts and Cricotracheal Resection (CTR) consists of resection of the stenosed segment of the airway with end-to-end anastomosis. The potential risks of these surgeries such as infection, restenosis and dehiscence in small pediatric airways justifies the need for coordinated and functional teamwork, particularly in the postoperative period.

The present study reports results in the treatment of subglottic stenosis during the time-frame of development of an airway team, critically revising aspects that may be involved in the failure or success of airway reconstruction in children in an attempt to identify predictive factors of success for this group.

## Patients and methods

A retrospective collection of information was approved by Institutional Ethics Committee board under n° 2.204.912.

An ongoing database of children undergoing open airway surgery during the 8-year period, from November 2008 to September 2017, coinciding with the development and training of an airway team, was retrospectively reviewed.

Patient demographics and information on cause of stenosis, grade of stenosis using Myer-Cotton classification,^8^ and presence of other airway anomalies that were defined as: uni- or bilateral vocal cord mobility impairment, tracheomalacia, supraglottic stenosis, vascular compression of the trachea were considered. Surgical data such as type of surgery performed, use of stents, type of stents, reoperations, need for postoperative endoscopic procedures and complications were also collected. Airway anomalies and surgical details were prospectively collected from surgical protocols completed during diagnostic endoscopic procedures and at the time of surgery.

In the past 5 years preoperative assessments have been established following a protocol with detailed mapping of the airway: grade of subglottic stenosis, mobility of vocal cords, distance from vocal cords to stenosis, number of tracheal rings from stenosis to tracheostomy, number of healthy tracheal rings from tracheostomy to carina and presence of malacic segments of the airway. This “airway mapping” is performed exclusively by airway endoscopy. Image studies are only ordered if there is suspicion of external tracheal compression or congenital tracheal malformation. Grade of stenosis and vocal cord mobility with restriction of interarythnoid space either by paralysis or scar tissue are the most important factors taken into account when deciding on which surgical procedure to perform (LTP or CTR). Nevertheless, other factors such as distance from vocal cords to SGS, from SGS to tracheostomy and presence of suprastomal collapse can also influence decision making. Mostly Grade II and lower Grade III stenosis will be treated with LTP and higher Grade III and Grade IV stenosis will be treated with CTR. In the case of Grade IV stenosis with a compromise of the posterior glottic space an extended CTR is indicated. Alongside mapping of the airway, cultures of tracheal secretions are collected a week before surgery and antibiotic planning as well as the proposed surgical procedure is discussed in a joint meeting with the intensive care staff (physicians and nurses). A discussion on the best sedation strategy and surgical staging depending on the child's age, extent of resection, comorbidities and psychological status is also part of the preoperative plan with the intensive care unit; adjustments are made accordingly in the postoperative period depending on additional intra-operative findings. Before the actual scheduling of the surgical procedure all children are evaluated by pediatric specialties involved in the control of comorbidities (pediatric pulmonology, cardiology, gastroenterology in most cases). Digestive endoscopies and chest CT's are not ordered routinely as described by some airway teams and are performed according to patient's symptomatology and airway findings during diagnostic endoscopies.

**Figure 1:**
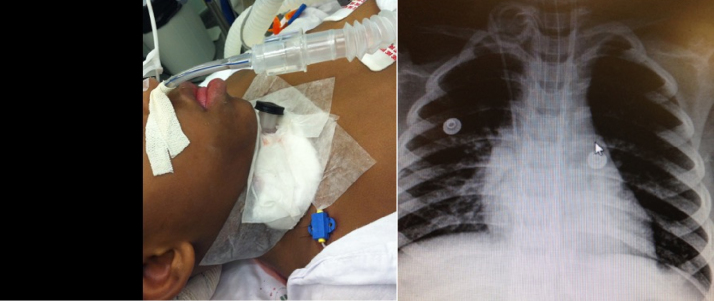
Illustration of patient submitted to hybrid technique with nasotracheal tube in place and capped tracheostomy cannula. On the right, a chest X-ray showing both nasotracheal tube and the 3.5 tube through the tracheostomy in place.

Surgeries were also described as single, double or hybrid technique. When the surgery was performed as a single-stage (SS) procedure the tracheostomy was resected during the airway reconstruction. When a tracheostomy was left in place or repositioned distally to the reconstructed airway this is called a double-stage (DS) procedure. The hybrid laryngotracheal reconstruction (LTR)^9^ refers to a DS procedure while leaving a small tracheostomy distal to the reconstruction and a nasotracheal tube simultaneously at the end of the surgery as a short term stent (Fig. 1). The advantage of this is use of a nasotracheal tube to stent the airway as one would with a single stage procedure and at the same time assure a safe airway after extubation with a tracheostomy. In these cases, patients were ventilated through an age-appropriate nasotracheal tube during the first 7–10 days while a small 3.5 tracheostomy cannula or a 3.5 tube with adjusted length was left capped (Fig. 1). After control endoscopy and extubation the tracheostomy was used (or intermittently capped) until a safe, epithelized airway was achieved. Double-stage surgery was chosen when there was a need for long-term stenting, generally due to glottic involvement.

Indication for postoperative dilatation was based on the finding of airway scar contraction or restenosis at the suture or graft site seen on follow-up endoscopy performed after 20 days postoperatively.

Cases with incomplete data and less than a 3-month follow-up were excluded from this report.

Descriptive analysis such as means with standard deviations, medians with inter-quartile ranges and frequencies or percentages for categorical variables was used to describe demographics and clinical characteristics. Correlation between demographic and surgical data and patient outcome was performed using Fischer and Mann–Whitney exact tests. Therapeutic success was defined as decannulation without noticeable noisy breathing, restrictive exertional dyspnea or repeated visits to healthcare centers for respiratory distress. Logistic regression models were used to determine whether age at the time of surgery, comorbidities, need for postoperative dilation, initial grade of stenosis, presence of other airway anomalies, fever in the postoperative period could be predictors for decannulation. The log rank Kaplan–Meier curve was used to compare results of LTP and CTR procedures throughout time. Decannulation rates were described as overall and operation specific, in this case taking into consideration only the first open airway procedure to which patients were submitted. Significance was declared at >0.05 and SAS 9.4 (SAS Institute, Cary, NC) was used to conduct all analyses.

## Results

**Figure 2:**
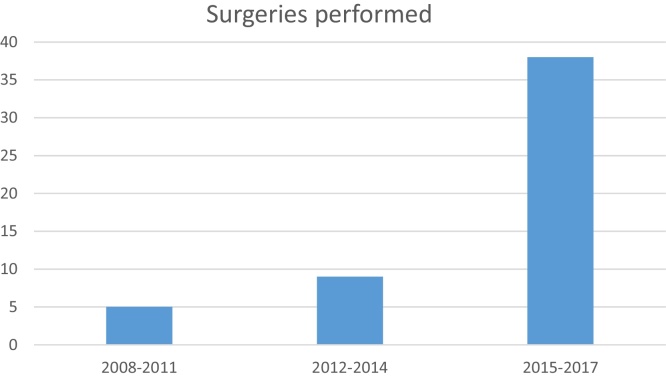
Number of reconstruction surgeries performed from 2008 to 2017.

In the past eight years 43 children underwent 52 open airway reconstructions. These were 50% laryngotracheoplasties (LTP) 50% cricotracheal resections (partial cricotracheal resections and extended cricotracheal resections). Patients with initial Grade II SGS were treated 87.5% with LTP while only 43.3% of the Grade III cases and 20% of the Grade IV underwent LTP. Progressive organization of the airway team coincided with an increase in the number of surgeries performed throughout the years (Fig. 2).

Patient demographics and type of surgeries performed can be appreciated on Table 1. Decannulation rates reported on Table 1 refer to those achieved per patient (final) considering the first surgery the patient was underwent. Operation-specific decannulation rates can be appreciated on Table 2.

**Table 1:** Demographic data and decannulation rates after first airway procedure and after revision surgery according to different grades of stenosis and type of first surgery performed for all 43 patients.

Demographic and clinical characteristics	Total patients (%)	Decannulation rates first surgery/final	*p*-Value
*Gender*	25 (58%) male		
*Comorbidities*	24 (55.8%)		
Prematurity	13 (30.2%)		
Lung disease	16 (37.2%)		
Cardiac disease	5 (11.6%)		
CF malformations	5 (11.6%)		
GERD	5 (11.6%)		
Other airway alterations/dysfunctions	15 (35%)		0.72

*Initial grade of SGS*			0.008
II	8 (18.6%)	100%	
III	30 (69.8%)	75.8%/90%	
IV	5 (11.6%)	25%/40%	

*Type of surgery*			0.78
LTP AG	9 (20.9%)	77.7%/77.7%	
LTP PG	7 (16.2)	85.7%/85.7%	
LTP AG + PG	5 (11.6%)	60%/100%	
PCTR/EPCTR	22 (51.1%)	68.1%/86.3%	

SGS, subglottic stenosis; LTP, laryngotracheoplasty; AG, anterior graft; PG, posterior graft; PCTR, partial cricotracheal ressection; EPCTR, extended partial cricotracheal ressection; GERD, gastro-esophageal reflux disease; CF, craniofacial; PCTR, partial cricotracheal resection; EXPCT, extended partial cricotracheal resection.

**Table 2:** Operation-specific decannulation rates.

Operation	Operation-specific decannulation rates (successful/total)
LTPAG	76.9% (10/13)
LTPPG	85.7% (6/8)
LTPAPG	75% (3/4)
PCTR	62.9% (17/27)
EPCTR	50% (1/2)
SS	83.3% (5/6)
DS	61.9% (13/20)
Hybrid LTR	85.1% (22/26)

LTPAG, laryngotracheoplasty with anterior graft; LTPPG, laryngotracheoplasty with posterior graft; LTPAPG, laryngotracheoplasty with anterior and posterior graft; PCTR, partial cricotracheal resection; EXPCT, extended partial cricotracheal resection; SS, single-stage; DS, double-stage; LTR, laryngotracheal reconstruction.

Of the 52 surgeries performed only 6 were SS, 20 where DS and 26 were performed using the hybrid technique (Table 2). Of the 20 DS procedures, long-term stenting (over 2 weeks) was used in 18. Stents were 12 LT molds® and 6 adapted T tubes. No statistical difference was observed between hybrid and double-stage surgery regarding decannulation (*p* = 1.0).

Etiology of SGS was tracheal intubation in all patients except two who had Type III laryngeal membranes. All patients except one had a tracheostomy at the time of surgery.

Initial grade of SGS showed a statistically significant correlation with successful outcome at the first operation (*p* = 0.0085).

Mean age at surgery was 4 years (average 4.59; 1–11.26 SD = 2.29). No correlation was found between age at which surgery was performed and outcome (Table 3). Over half (55.8%) of the patients presented at least one comorbidity. Logistic regression showed that children with comorbidities presented a 2.5 chance of surgical failure (95% IC 1.2–4.9, *p* = 0.0067) as can be appreciated on Table 3.

**Table 3:** Logistic regression for correlation of risk factors related to surgical outcome.

	OR	95% IC	*p*-Value
Grade IV vs. Grade II stenosis	19.1	–	0.014
Grade III vs. Grade II stenosis	3.831	–	0.2223
Grade III vs. Grade IV stenosis	10.5	1.014–108.755	0.0487
Comorbidities	2.536	1.294–4.969	0.0067
Dilatations	3.3	0.785–13.879	0.1033
Fever	0.913	0.193–4.330	0.9088
Age	1.025	0.768–1.369	0.8659
Other airway anomalies	1.429	0.361–5.656	0.6114

When considering the first surgery performed, type of surgery did not influence surgical outcome (Table 1) (*p* = 0.78). Operation-specific success rates can be appreciated on Table 2.

**Figure 3:**
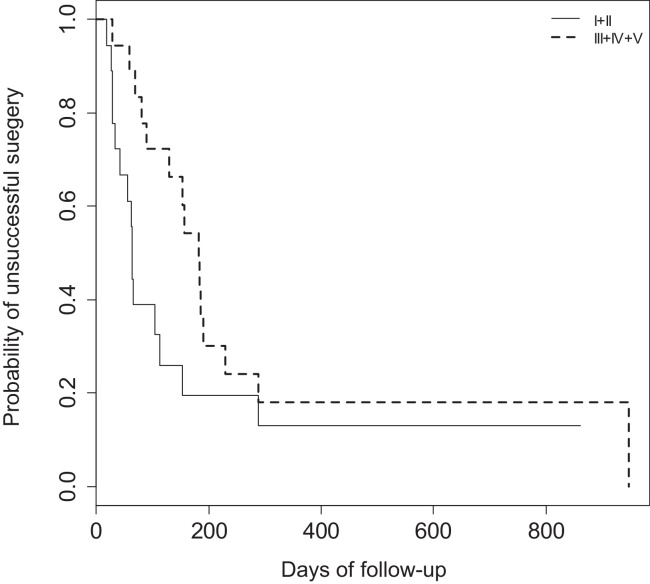
Kaplan–Meier comparing resection surgeries (PCTR and EPCTR) and expansion surgeries (LTP). I + II = PCTR and EPCTR; III + IV + V = LTPAG + LTPPG + LTPAPG.

When comparing expansion surgeries (LTP's) and resection surgeries (CTR) results throughout time using a log-rank Kaplan–Meier curve no statistical difference (*p* = 0.0772) was found (Fig. 3).

Other airway anomalies such as uni- or bilateral vocal cord mobility impairment, associated supraglottic stenosis, tracheomalacia and vascular compression of the trachea were present in 34.8% (Table 1) of the cases but were not predictive of surgical outcome (*p* = 0.726). Of the 15 cases with other airway anomalies, 11 were limited vocal cord abduction due to vocal cord palsy or scar tissue involvement of the posterior glottis and four were tracheomalacia, one of which was later associated with vascular compression of the trachea.

Fever was present in 76.7% of the patients for an average of 5 days in the postoperative period.

Postoperative dilatation was performed in over a third of patients (34.15%). Postoperative dilatation was not associated with the outcome (*p* = 0.103) but logistic regression showed a 3.3 bigger chance of failure when dilatation was performed. A greater number of dilatations were observed for Grade IV SGS than for Grade II and III SGS (average of 4.3, 1.5 and 1.3 respectively).

The overall decannulation rate was 86% (37/43) during the study period for this group of children and 72% (31/43) were successfully decannulated after one single procedure. Seven children were submitted to 11 revision or salvage surgeries due to restenosis or segmental malacic deformities that prevented decannulation.

The 6 children who were not decannulated during the study period consisted of two deaths before decannulation, one child who is currently scheduled for salvage surgery, one currently using a small tracheostomy due to persistent tracheomalacia and obstructive sleep apnoea and, two undergoing further investigation for comorbidity control before salvage surgery.

The two deaths that occurred were related to postoperative complications; one on the 9th postoperative day after accidental extubation of a single-stage PCTR and dehiscence caused by attempted reintubation and another due to infection and sepsis three weeks after a double-stage PCTR. Both complications occurred in the first three-year period after reconstructive surgeries started to be performed, that is at the beginning of establishment of a multidisciplinary preoperative and postoperative planning.

Time interval between surgery and decannulation was 3.6 months (min: 0.63; max: 31.57 months) and median follow-up was 7.5 months (min: 0.27; max: 71.77 months).

## Discussion

Overall decannulation rates for reconstructive airway surgeries in children vary largely depending of initial grade of stenosis. The most important airway teams performing the largest numbers of surgeries report overall decannulation rates for Grade III stenosis from 78%^10^ to 79%^11^ and from 50%^10^ to 88%^11^ for Grade IV stenosis. In the present study overall decannulation rate was 86% during the study period with 72% of children being decannulated after one single open airway procedure and over 80% of the cases represented Grade III and IV stenosis. This is comparable to other reports for the pediatric population^1^, ^3^, ^12^, ^13^ and also to adult populations being treated for airway stenosis.^14^ The relatively worse results obtained for the Grade IV cases in this series when compared to the literature may have been biased by the relative low number of Grade IV cases and also by the fact that this is a relatively new airway team. Undoubtedly these are the most challenging cases, particularly when the stenotic segment presents very cranially close to the vocal cords, demanding high sutures and a complete laryngofissure. In agreement with previous authors age was not predictive of surgical failure. Appropriate surgical selection and preoperative assessment is more important than patient age in determining the feasibility and success of airway reconstruction. The risk to a small child with complete upper airway obstruction at home with a tracheostomy cannot be taken for granted. Other authors have demonstrated that reconstructive airway surgery is a safe procedure even in very small children.^4^, ^5^, ^15^ Ikonomidis et al., in their series of 36 children weighing less than 10 kg who underwent partial cricotracheal resection demonstrated no significant difference in outcome of this group when compared to a group of older children.

As expected, grade of stenosis correlated to worse surgical results. Padia et al.^16^ in their recent systematic review/meta-analysis reported that even when comparing single and double-stage procedures worse success rates were only observed at higher grades of stenosis. In the current series over 80% of the cases were Grade III and IV stenosis and this may also account for a relatively high number of cases (7 patients, 16.2%) undergoing salvage surgery.

Although the type of surgery performed (Table 1) did not significantly influence outcomes, considering that worse cases (Grades III and IV) were treated preferably with CTR, one may argument that this would be a better procedure than LTR. On the other hand, LTP and CTR did not predict significant outcomes in this group. It is possible that the option for a procedure with greater mortality (CTR) is not justified over time. Nevertheless, Grade III SGS varies greatly (71–99%) and scar tissue formation varies from one patient to the other. The surgeon's personal preference and experience is crucial in the selection of these procedures and adjustments should be made according to the characteristics of the airway team and hospital setup particularly in the postoperative period. Availability of appropriate airway stents in the choice between an LTP with anterior and posterior grafting and a longer stenting period and a CTR for example must also be considered.

The role of comorbidities in surgical failures has been addressed by several authors^1^, ^17^ and Monnier^18^ that describes a new staging system taking into consideration the presence of glottic abnormalities and presence of comorbidities to predict surgical outcome. This was confirmed by the findings in this cohort with logistic regression confirming the greater risk of failed decannulation in such cases. Despite being the case in a third of the cases in this cohort airway abnormalities were not predictive of surgical failure. This may possibly have been less of an issue due to the fact that diagnosis was made preoperatively; this was taken into account when considering the need of long-term stenting, follow-up endoscopies and postoperative assessment. The fact that over half of the patients presented comorbidity and these are predictive of surgical outcome is indicative that multispecialty teamwork can significantly contribute to better surgical outcome.

Single-stage surgeries have been reported as being more successful than double-stage surgeries^17^ due to less tracheal contamination. Padia et al.^16^ in a recent meta-analysis found higher success rates for single stage surgeries when compared to double stage surgeries (93.2% vs. 83.7% respectively) however when controlling for stenosis severity no difference was observed. When long-term stenting is required (over 2 weeks) usually due to complex or multi-level stenosis double-staging is mandatory. This may be a bias when comparing results for single and double-stage procedures since double-stage procedures are usually performed in more complex patients. Smith et al.^19^ recently compared outcomes in reconstructive airway surgery with short time versus long-term stenting and reported better decannulation rates with long-term stenting. In the current series DS results were worse (60%) while SS (83.3%) and hybrid LTR (85.1%) had comparable results. These results nevertheless may be biased since 18 out of 20 of the DS procedures were also procedures requiring long-term stenting due to multilevel laryngeal scarring translating more complex laryngeal stenosis. The decision for single or double staging a surgery must take into account the resources and setup available for the airway team^17^ and this is particularly true considering the reality of intensive care set up in our country. Raol et al.^13^ described similar decannulation rates for the hybrid LTR (76.9%) although in their casuistic this was similar to the decannulation rate with DS procedures.

About one third of reconstructive surgeries demanded airway dilatation in the postoperative period before or after decannulation. This is also described by other authors and therefore it is important that airway surgeons are trained both in endoscopic procedures and open airway procedures, since there may be a need for both procedures in the same patient. It is particularly important that families understand the need for follow-up procedures following airway reconstruction since the scarring condition can only be accessed through endoscopy.

Although other airway anomalies occurred in over a third of the cases the fact that worse decannulation rates were not associated with these findings differs from what has been reported by the most important groups worldwide.^1^, ^2^ Nevertheless, this information may be biased by the limited number in this cohort.

Despite the overwhelming number of children waiting for reconstructive airway surgery in our hospital it is our belief that an increasing number of surgeries can only be achieved through a functional airway team that communicates and interacts, since the preoperative period minimizing risks of operating on children that are at greater risk for surgical failure and morbidity through the postoperative period until decannulation is achieved. Surgeons must be skilled in endoscopic diagnostic, treatment and staging procedures and also master all reconstructive techniques. The value of documentation and feedback to the entire team involved in treating these patients cannot be taken for granted.

Comparing success rates in airway reconstruction surgery is difficult since acquired scarring of the larynx can involve supraglottic, glottis and subglottic segments of the larynx in very distinct forms; individual inflammatory response is not yet completely understood and may account for poorer results. Individualized evaluation when preparing for airway reconstruction and also in the evaluation of airway response to manipulation is essential and has been mentioned by other authors.^3^ Limitations of this case series are similar to other case series, that is, in such diverse pathology very large sample sizes are required to allow for patient stratification into comparable subgroups. Continued documentation and reports of results on a larger number of patients may confirm these observations.

Future studies may focus on long-term follow-up of pediatric airway reconstruction reporting quality of voice and communication skills and the presence of residual exertional airway dysfunction, although the burden to a child with a tracheostomy is probably the most impactful issue for these families.

## Conclusion

Overall decannulation rate was 86%. Failures were associated with higher grades of stenosis and presence of comorbidities but not with patient age or concomitant airway anomalies.

## Conflicts of interest

The authors declare no conflicts of interest.
